# Hybrid *Broussonetia papyrifera* Fermented Feed Can Play a Role Through Flavonoid Extracts to Increase Milk Production and Milk Fatty Acid Synthesis in Dairy Goats

**DOI:** 10.3389/fvets.2022.794443

**Published:** 2022-03-11

**Authors:** Mengjie Zhao, Dongliang Lv, Jingcao Hu, Yonglong He, Zhi Wang, Xinyu Liu, Benkang Ran, Jianhong Hu

**Affiliations:** College of Animal Science and Technology, Northwest A&F University, Yangling, China

**Keywords:** dairy goats, hybrid *Broussonetia papyrifera*, goat mammary epithelial cells, flavone, triglyceride

## Abstract

In order to explore the effect of hybrid *Broussonetia papyrifera* fermented feed on milk production and milk quality of dairy goats, and to compare with alfalfa hay, three dairy goat diets were designed based on the principle of equal energy and equal protein. The goats in the control group were fed a basic TMR diet (CG group), and the other two groups were supplemented with 10% alfalfa hay (AH group) and 10% hybrid *B. papyrifera* fermented feed (BP group). The results showed that the dry matter intake and milk production of BP group increased significantly. The total amount of amino acids and the content of each amino acid in the milk of AH group and BP group were lower than those of CG group. The content of saturated fatty acids in the milk of BP group decreased while the content of unsaturated fatty acids increased. The contents of prolactin, estrogen and progesterone in BP goat serum were generally higher than those of AH goat and CG goat. Subsequently, this study separated and cultured mammary epithelial cells from breast tissue, and added flavone extracted from the leaves of hybrid *B. papyrifera* and alfalfa to their culture medium for comparison, which is one of their important bioactive components. The results showed that low-dose alfalfa flavone (AH) and hybrid *B. papyrifera* flavone (BP) can increase cell viability. They also can increase the accumulation of intracellular triglyceride and the formation of lipid droplets. Both AH flavone and BP flavone significantly up-regulated the expression of genes related to milk fat synthesis, including genes related to fatty acid *de novo* synthesis (*ACACA, FASN*, and *SCD1*), long-chain fatty acid activation and transport related genes (*ACSL1*), and genes related to transcription regulation (*SREBP1*). The three genes related to triglyceride synthesis (*DGAT1, DGAT2*, and *GPAM*) were all significantly increased by BP flavone. Both AH flavone and BP flavone significantly increased the protein expression of progesterone receptor and estrogen receptor in mammary epithelial cells but had no effect on prolactin receptor.

## Introduction

Goat milk is more similar to human milk, the curd is softer, and the proportion of small milk fat globules is higher (1). Compared with cow's milk, it is easier to absorb and has the characteristics of anti-allergic and high protein and mineral content (2). Goat milk contains protein, lactose, fat, inorganic elements, amino acids, and enzymes, as well as rich vitamins, minerals, and inorganic salts (3). The total amount of amino acids in goat milk are about 1–2%, and the content of essential amino acids is about 45% of the total amount of amino acids (4). Compared with cow's milk, goat milk can provide higher content of lysine, leucine, valine, and threonine (5). Supplementing marine oils or essential oils in the diet of lactating animals will increase the concentration of beneficial fatty acids of milk (6). And some polyunsaturated fatty acids (PUFA) are very important, such as linolenic acid can prevent arrhythmias and sudden cardiac death (7). Prolactin (PRL) is a protein hormone secreted by the eosinophils of the anterior pituitary gland. Its main role is to regulate the lactation function of goats and promote lactation (8). PRL can cooperate with estrogen (E) and progesterone (P_4_) to promote the development of the breast system (9). There are two sources of triglycerides (TAG) in goat milk: fatty acid biosynthesis and esterification in the mammary glands, and lipids enter the mammary glands from plasma (10). Short-chain (C <6) and medium-chain fatty acids (C6–C16) are generally synthesized *de novo* under the control of several key factors and enzymes including sterol regulatory element binding protein 1 (SREBP1), acetyl-CoA carboxylase α (ACACA), fatty acid synthase (FASN), and stearoyl-CoA desaturase (SCD), while Long-chain fatty acids (C>16) are absorbed from plasma through fatty acid binding protein 3 (FABP3), acyl-CoA synthetase long-chain family member 1 (ACSL1) and solute carrier family 27 member 6 (SLC27A6) (11, 12). These fatty acids in the mammary gland can be desaturated by SCD, and then can be secreted into goat milk in the form of fat globules by diacylglycerol O-acyltransferase 1 (DGAT1), diacylglycerol O-acyltransferase 2 (DGAT2), and glycerol-3-phosphate acyltransferase 1 (GPAM) (13). SREBP1 promotes the expression of fatty acid biosynthesis-related genes such as *ACACA* and *FASN* (14), and peroxisome proliferator activated receptor γ (PPARγ) also plays a role in regulating milk fat synthesis (15).

The research of diet nutrition level on goat mammary gland function is an important part of the relationship between food nutrients and body metabolism. Existing results has shown that changes in diet have a critical impact on goat milk amino acids (16), fatty acid composition and content (17), and lactation-related hormones (18). Interestingly, it also interferes with the expression of genes related to breast fatty acid metabolism (19). Protein feed is still an important feed source due to the shortage of high-quality feed resources. Alfalfa has a long history of cultivation and is one of the largest perennial leguminous pastures in the world. It has the characteristics of rich nutrition, good palatability, and easy digestion. The crude protein content of alfalfa cut at the first flowering stage can reach about 18% (20). Hybrid *Broussonetia papyrifera* has the advantages of strong stress resistance and high resistance to diseases and insect pests. The leaves of hybrid *B. papyrifera* have a high protein content of which exceeds 20% and can be used directly as livestock feeds or as a raw material for a total mixed ration (TMR) (21). Now, by making hybrid *B. papyrifera* fermented feed, it can not only preserve a large amount of vitamins, but also make it taste sour, sweet and mellow (22). It has been reported that flavone extracted from alfalfa and hybrid *B. papyrifera* are plant secondary metabolites with many functions, such as pigmentation, antimicrobial activity and antioxidant activity (23, 24). Moreover, the estrogenic effects of flavone have been confirmed in many experiments (25, 26).

In this experiment, 10% alfalfa and 10% hybrid *B. papyrifera* fermented feed were added to the milk goat TMR diet to measure milk components, milk amino acids, fatty acid composition, and lactation-related hormones. The flavone extracted from alfalfa and hybrid *B. papyrifera* were added to the culture medium of goat mammary epithelial cells (gMECs) to explore the synthesis mechanism of fatty acids from the perspective of molecular biology. *In vivo* and *in vitro* experiments were used to explore the effects of adding hybrid *B. papyrifera* fermented feed to the TMR diet on goat milk quality and lactation performance, accumulating theoretical basis for improving goat milk production and nutritional quality.

## Materials and Methods

All animal experiments were approved by the Animal Protection and Utilization Committee of Northwest Agriculture and Forestry University (Yangling, Shaanxi).

### Animals and Diets

This experiment was carried out in the standard animal breeding farm of Shaanxi Aonike Guanzhong Dairy Goat Breeding Co., Ltd (Shaanxi, China). Eighteen Guanzhong dairy goats (100 ± 5 days of lactation) with good health and the same average initial weight (51.5 ± 4.6 kg) were selected, and the goats were randomly divided into three groups. There were six animals in each group, and each group ate one of three diets. The ration was formulated based on the recommendations of the NRC (2007) and follows the principle of equal nitrogen to provide them with sufficient energy, protein, vitamins, and minerals. Ingredients of the diets is shown in Table 1: (1) the diet contains 17% oat grass and 33% alfalfa hay (CG group); (2) the diet contains 7% oat grass and 43% alfalfa hay (AH Group); (3) the diet contains 10% hybrid *B. papyrifera* fermented feed, 33% alfalfa hay and 7% oat grass (BP group). The hybrid *B. papyrifera* fermented feed was produced and provided by Shaanxi Pangnong Ecological Agriculture Technology Co., Ltd (Shaanxi, China). The hybrid *B. papyrifera* was planted in Liuji Town, Fuping County, Xianyang City, Shaanxi Province. It was harvested in June 2020 and made into fermented *B. papyrifera* feed then opened for use in July. The feed ingredients were mixed using a horizontal feed mixer and fed in the manner of TMR. The experiment lasted for 8 weeks, and the pre-experiment was carried out in the first 2 weeks so that the goats gradually adapted. All goats were fed at 07: 00, 12: 00, and 19: 00 each day and they were free to drink water.

**Table 1:** Ingredients and nutrient level of the three experimental diets based on oat Grass, alfalfa hay, and hybrid *Broussonetia papyrifera*.

**Items**	**Treatment**		
	**CG**	**AH**	**BP**
**Ingredient (% of DM)**			
Hybrid *Broussonetia papyrifera* fermented feed	0.00	0.00	10.00
Alfalfa hay	33.00	43.00	33.00
Oat grass	17.00	7.00	7.00
Maize	28.00	29.00	30.00
Bran	7.00	6.00	5.00
Soybean meal	10.00	10.00	10.00
Soy flour	2.00	2.00	2.00
Soda	0.25	0.25	0.25
Salt	0.25	0.25	0.25
Premix	2.50	2.50	2.50
Total	100.00	100.00	100.00
**Nutritional level (%)**			
Dry matter	62.64	71.30	48.85
Net energy (MJ/kg)	3.61	3.63	3.66
Crude protein	18.80	18.50	18.90
Digestible crude protein	14.88	14.80	14.74
Ruminal bypass protein	3.92	3.70	4.16
Crude fat	3.20	2.80	3.20
Neutral detergent fiber	38.36	40.26	43.86
Acid detergent fiber	25.50	28.20	28.60
Lingnin	4.60	5.40	5.40
Calcium	0.96	0.99	1.13
Phosphorus	0.47	0.44	0.46
Ash	9.42	9.08	9.31

*DM, dry matter.*

*CG: The diet contains 17% oat grass and 33% alfalfa hay; AH: The diet contains 7% oat grass and 43% alfalfa hay; BP: The diet contains 10% hybrid Broussonetia papyrifera fermented feed, 33% alfalfa hay, and 7% oat grass*.

### Sample Collection and Assay

During the experiment, the feed samples were collected every day and kept at room temperature until the nutrients in the diet were analyzed. Milking was performed twice a day at 5:00 and 17:00 and the individual milk production was recorded. Record the dry matter intake (DMI) during feeding. The weight of each goat was weighed before the start of the experiment and on the last day of the experiment. Collect goat milk samples from each goat in the morning and evening on the last day of the week, mix each group of samples thoroughly and store at −20°C. Use a milk component analyzer to analyze milk protein, milk fat, non-fat solids, lactose, etc. Goat blood was collected on the last day of the week and the serum was separated and stored at −20°C.

### Chemical Analysis of Diet

The dry matter, net energy, crude protein, digestible crude protein, ruminal bypass protein, crude fat, neutral detergent fiber, acid detergent fiber, lignin, calcium, phosphorus, and ash in the diets were tested and shown in Table 1. All tests were completed by the laboratory of Yima Agriculture and Animal Husbandry Technology Co., Ltd (Inner Mongolia, China). Near-infrared spectroscopy (NIR) was used to detect the nutritional level of diets.

### Milk Amino Acid and Fatty Acid Analysis

Amino acid analyzer (membraPure GmbH, Germany) and gas chromatograph (Agilent Technologies, Shanghai, China) were used to quantitatively analyze the fatty acids and amino acids in milk. The collected goat milk was divided into two parts, one part was analyzed for amino acid composition, and the other part was analyzed for fatty acid composition. Use GB 5009.124-2016 “National Food Safety Standard Determination of Amino Acids in Foods” to detect amino acid, and use GB 5009.168-2016 “National Food Safety Standard Determination of Fatty Acids in Foods” to detect fatty acid.

### Detection of Hormone Content in Serum

Blood was collected from the jugular vein of dairy goats with a vacuum blood collection tube containing heparin sodium at 7 am on the last day of each week. Centrifuge at 3,000 rpm for 15 min to separate and obtain the serum, mix the serum of each group thoroughly. Different goat hormone enzyme-linked immunoassay kits (E, PRL, and P_4_) were used to detect the content of serum hormones. Briefly, serum was added to the microwells coated with monoclonal antibodies in sequence, and then combined with HRP-labeled hormone antibodies. After thorough washing, the substrate was added to develop color. The microplate reader (Bio Tek, USA) was used to measure the absorbance at a wavelength of 450 nm, and then the concentration of related hormones in the serum was calculated from the standard curve. The kits were purchased from Shanghai FANKEL Industrial Co., Ltd (Shanghai, China).

### Cell Isolation, Culture, and Treatment

The previous method (27) was used to isolate gMECs. The mammary gland tissue was collected by surgery and cut into small pieces of about 1 mm^3^, and then digested with 1 mg/mL collagenase (Solarbio, Beijing, China) at 37°C for 2 h. The digestion solution was filtered with nylon mesh, and the filtrate was centrifuged at 150 × g for 10 min. Cells were resuspended and inoculated in basic DMEM/F12 medium (Hyclone, US) containing 10% fetal bovine serum, 10 ng/mL epidermal growth factor 1, 100 U/mL streptomycin/penicillin and 0.3 mmol/mL hydrocortisone (Gibco,CA,USA). The cells were cultured at 37°C with 5% CO_2_ in a humid atmosphere. Then they were divided into 10 treatment groups, and treated with alfalfa flavone (0, 1, 2, 4, 8, and 16 μM) and hybrid *B. papyrifera* flavone (0, 1, 2, 4, 8, and 16 μM). All treatments were repeated 3 times. The cells were then incubated separately for 48 h for further testing. Alfalfa flavone was purchased from Shaanxi Lvqing Biological Engineering Co., Ltd (Shaanxi, China), and hybrid *B. papyrifera* flavone was purchased from Baoji Chenguang Biological Technology Co., Ltd (Shaanxi, China). They were both purified products with a purity of over 98%.

### Cell Viability Assay

Add 10 μL of CCK-8 solution (NCM Biotech, Suzhou, China) to the gMECs seeded in a 96-well-plate, and incubate at 37°C for 2 h to evaluate the gMECs activity. Then, use a microplate reader (Bio Tek, USA) to measure the absorbance at 450 nm.

### Assessment of Triglyceride Content and Oil Red O Staining

The TAG content was determined using a TAG detection kit (Applygen, Beijing, China). Briefly, the medium was removed, and the remaining cells were collected by washing with PBS. The cell samples were treated with RIPA lysis buffer (Beyotime, Nanjing, China). The content of TAG in the supernatant was determined with a kit. The protein concentration in the supernatant was determined with the BCA protein determination kit (Thermo Fisher Scientific, Waltham, MA, USA). The content of TAG was determined by normalization to the total protein of each sample.

Inoculate gMECs in 6-well-plates. Aspirate the culture medium and wash 4 times with cold PBS, and fix with 10% paraformaldehyde at 4°C for at least 40 min. Use 0.5% oil red O dye solution (0.05 g oil red O dry powder dissolved in 10 ml isopropanol at room temperature. Filter with 0.2 μm filter 37°C for 20–30 min). After staining, wash with PBS twice. Then observe under a fluorescence microscope. Finally, use ImageJ software to measure the percentage of area covered by Oil Red O. Oil red O dry powder was purchased from Beijing Boao Tuoda Technology Co., Ltd (Beijing, China).

### RNA Extraction and Real-Time Quantitative PCR

Wash the gMECs twice with PBS. Use RNA extraction kit to isolate total RNA from gMECs, and use DNAse to remove DNA contamination. The purity of RNA (A260/A280) was measured with a spectrophotometer for all samples, and the purity was 1.8–2.0, indicating that the sample was of high purity. And use ethyl sulfide bromide staining agarose gel to detect the integrity of RNA. Use reverse transcription kit to get the first strand of cDNA from total RNA. Dilute cDNA 1:5 with DNase/RNase free water. Design primers based on the cDNA sequence. See Table 2 for primers. Detect the transcript abundance of target genes and internal reference genes by qPCR. Perform qPCR according to the kit SYBR Green Real-Time PCR Master Mix. Using Bó Lè instruments, the specific procedures include initial denaturation at 95°C for 1 min, 40 amplification cycles, including denaturation at 95°C for 15 s, annealing at primer-specific temperature (58–61°C) for 15 s and at 72°C extends for 20 s. Ethylene bromide was used for melting curve analysis and qPCR product monitoring to evaluate amplification specificity. The mRNA levels of all samples were normalized to the value of the internal control (*GAPDH*), and the results were expressed as the fold change of the threshold period (Ct) value relative to the control group, using the 2-ΔΔCt method. All kits were purchased from Beijing Tiangen Biochemical Technology Co., Ltd (Beijing, China).

**Table 2:** The information of primers for qRT-PCR.

**Gene**	**GenBank ID number**	**Primer sequence (5′to 3′)**	**Length (bp)**	**Reference**
DGAT1	*DQ380249.1*	*F: CCACTGGGACCTGAGGTGTC*	101	(28)
		*R:GCATCACCACACACCAATTCA*		
DGAT2	*HM566448.1*	*F:CATGTACACATTCTGCACCGATT*	100	(29)
		*R:TGACCTCCTGCCACCTTTCT*		
GPAM	*AY515690*	*F: ATTGACCCTTGGCACGATAG*	188	(29)
		*R:AACAGCACCTTCCCACAAAG*		
SREBP1	*HM443643.1*	*F:CTGCTGACCGACATAGAAGACAT*	81	(28)
		*R: GTAGGGCGGGTCAAACAGG*		
PPARA	*XM_005681211.1*	*F:CGGTGTCCACGCATGTGA*	56	(29)
		*R:TCAGCCGAATCGTTCTCCTAAA*		
PPARG	*HQ589347.1*	*F:CCTTCACCACCGTTGACTTCT*	145	(29)
		*R:GATACAGGCTCCACTTTGATTGC*		
FABP3	*NM_001285701.1*	*F: GATGAGACCACGGCAGATG*	120	(28)
		*R: GTCAACTATTTCCCGCACAAG*		
ACSL1	*BC119914*	*F:GTGGGCTCCTTTGAAGAACTGT*	120	(29)
		*R:ATAGATGCCTTTGACCTGTTCAAAT*		
SLC27A6	*CK772265.1*	*F:CAACTTGCTCATAAACTTTTTCCAAG*	101	(29)
		*R:TGGTGTGGTTGTGCCAGGT*		
ACACA	*XM_005693156.1*	*F:CTCCAACCTCAACCACTACGG*	171	(28)
		*R:GGGGAATCACAGAAGCAGCC*		
FASN	*DQ915966.3*	*F:GGGCTCCACCACCGTGTTCCA*	226	(28)
		*R:GCTCTGCTGGGCCTGCAGCTG*		
SCD1	*GU947654*	*F:CCATCGCCTGTGGAGTCAC*	257	(28)
		*R:GTCGGATAAATCTAGCGTAGCA*		
GAPDH	*XM_005680968.3*	*F: TCAAGAAGGTGGTGAAGCAG*	249	This manuscript
		*R: AAGGTAGAAGAGTGAGTGTCGC*		

### Western Blot Analysis

The gMECs was lysed on ice with a cell lysis buffer containing RIPA and PMSF (100:1) for 30 min to obtain a total protein lysate. Determine the total protein concentration with the BCA detection kit, and then boil the sample buffer at 100°C for 10 min. The protein samples (30 μg protein per channel) were electrophoresed in 8–10% sds-polyacrylamide gel. The protein was separated by PAGE and transferred to a polyvinylidene fluoride membrane, and then the protein was transferred to a nitrocellulose membrane and incubated with the indicated primary antibody. Incubate the blot with the primary antibody at 4°C overnight. Primary antibodies: PRLR (proteintech, 67292-1-lg, 1:1,000), ER (proteintech, 21244-1-AP, 1:1,000), PR (proteintech, 25871-1-AP, 1:1,000). Finally, after washing with TBST, incubate with alkaline phosphatase-labeled secondary antibody (diluted with 1:10,000 TBST) at 37°C for 1 h. The reaction protein was visualized with chemiluminescence (ECL) reagents and quantified with ImageJ software. The protein level was normalized to Tubulin.

### Statistical Analysis

Each experiment was repeated at least three times. When only one factor affected, one-way analysis of variance (ANOVA) of SPSS19.0 statistical software and GraphPad software were used to analyze the difference between the data, and the data was shown as the means and SEM. When the two factors of different diets and feeding time affected, the general linear model (GLM) of SPSS19.0 statistical software was used to conduct multi-variance ANOVA of DMI and milk production, and the data shown as means and SEM. *p* < 0.05 is considered to be significant, and *p* < 0.01 is considered to be extremely significant.

## Results

### Changes in DMI and Body Weight of Goats

**Figure 1:**
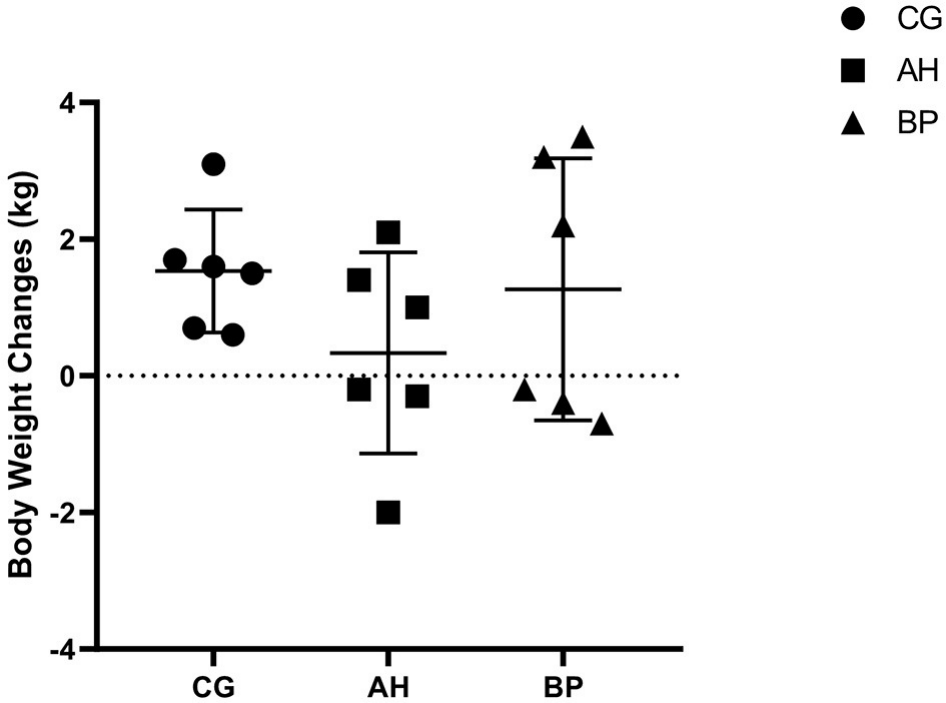
The changes of body weight of goats before and after experiment.

As shown in Table 3, in the 1st week of the experiment, there was no difference in the DMI of the three groups of goats. As time went on, the DMI began to increase. The DMI of goats in the BP group was higher than that of the CG group in the 4th week (*p* < 0.01) and the 6th week (*p* < 0.05). And the DMI of the goats in the AH group was lower than the CG group in the 3rd week (*p* < 0.05). The DMI in BP goats was higher than that in AH group in the 3rd week (*p* < 0.01), 4th week (*p* < 0.01), 5th week (*p* < 0.05), and 6th week (*p* < 0.01). Moreover, the interaction between treatments of diets and feeding time had an impact on DMI (*p* < 0.01). The weight changes of the three groups of dairy goats before and after the feeding period only fluctuated between 0.2 and 3.5 kg, and the weight of individual goats slightly decreased (Figure 1).

**Table 3:** The changes in DMI of goats.

**Items**	**Time point**	**CG**	**AH**	**BP**	**SEM**	***P*** **-value**		
						**Treatment**	**Time**	**Treatment × time**
DMI (kg/d)	w1	2.31^C^	2.28^C^	2.38^C^	0.02	0.47	_	_
	w2	2.36^C^	2.34^B^	2.38^B^	0.01	0.12	0.02	0.16
	w3	2.49^Ba^	2.43^Bb^	2.62^Ba^	0.02	0.01	<0.01	<0.01
	w4	2.56^ABb^	2.45^ABb^	2.71^Aa^	0.03	<0.01	<0.01	<0.01
	w5	2.65^ABab^	2.55^Ab^	2.69^Aa^	0.03	<0.01	<0.01	<0.01
	w6	2.63^Ab^	2.55^Ab^	2.70^Aa^	0.02	<0.01	<0.01	<0.01

*DMI, dry matter intake. w1–w6 represents the 1st week to the 6th week.*

*Mean values without common superscript (a and b) differ significantly among CG, AH, and BP groups (p < 0.05). Mean values without common superscript (A, B, and C) differ significantly between w1 and w6 (p < 0.05)*.

### Milk Production and Composition

As shown in Table 4, feeding time had no effect on the changes in milk production (*p* > 0.05), but we have observed that as the experiment progresses, the milk production first rises and then falls. The milk production of BP group was higher than that of CG group in the 2nd week (*p* < 0.05), 5th week (*p* < 0.01), and 6th week (*p* < 0.01). In the 2nd week (*p* < 0.05), the 4th week (*p* < 0.01), the 5th week (*p* < 0.01), the 6th week (*p* < 0.01), the milk production of BP group was higher than AH group. There were no differences in the milk protein, milk fat, non-fat solids and lactose content of the three groups of goat milk (*p* > 0.05), but the milk fat rate of the BP group was higher than that of the CG group (Table 5).

**Table 4:** The changes in milk production of goats.

**Items**	**Time point**	**CG**	**AH**	**BP**	**SEM**	***P*** **-value**		
						**Treatment**	**Time**	**Treatment × time**
Milk production (kg/d)	w1	1.78	1.85	2.25	0.08	0.40	_	_
	w2	1.97^b^	1.87^b^	2.49^a^	0.08	0.02	0.58	0.32
	w3	1.96	2.03	2.39	0.07	0.38	0.42	0.41
	w4	1.97^ab^	1.81^b^	2.32^a^	0.04	<0.01	0.22	0.31
	w5	1.98^b^	1.67^b^	2.32^a^	0.06	<0.01	0.34	0.10
	w6	1.71^b^	1.77^b^	2.14^a^	0.07	<0.01	0.53	0.06

*Mean values without common superscript (a and b) differ significantly among CG, AH, and BP groups (p < 0.05)*.

**Table 5:** Milk composition of goats.

**Items (%)**	**CG**	**AH**	**BP**	**SEM**	***P*-value**
Milk fat	3.44	3.33	3.56	0.28	0.724
Not fat solid	8.59	8.31	7.90	0.41	0.295
Lactoprotein	3.18	3.13	3.08	0.20	0.878
Lactose	4.57	4.59	4.52	0.03	0.072

*Mean values without common superscript (a and b) differ significantly among CG, AH, and BP groups (p < 0.05)*.

### Milk Amino Acid and Fatty Acid Composition

The amino acid composition and content of goat milk were shown in Table 6. Compared with CG group, the content of each amino acid and the total amount of amino acids in the AH group was reduced, and the BP group was also lower than the CG group (*p* > 0.05).

**Table 6:** Amino acids composition in the milk.

**Amino acids (g/100 g)**	**CG**	**AH**	**BP**	**SEM**	***P*-value**
Asp	0.34	0.27	0.29	0.03	0.106
Thr	0.21	0.18	0.18	0.04	0.653
Ser	0.27	0.23	0.25	0.03	0.333
Glu	1.06	0.93	0.96	0.17	0.731
Pro	0.32	0.31	0.29	0.38	0.670
Gly	0.09	0.07	0.07	0.02	0.453
Ala	0.14	0.10	0.11	0.02	0.256
Val	0.36	0.31	0.30	0.07	0.646
Met	0.12	0.10	0.11	0.03	0.880
Tle	0.19	0.17	0.16	0.03	0.630
Leu	0.47	0.39	0.40	0.12	0.776
Tyr	0.15	0.12	0.13	0.03	0.690
Phe	0.22	0.19	0.19	0.06	0.819
His	0.16	0.14	0.14	0.04	0.816
Lys	0.38	0.33	0.33	0.09	0.805
Arg	0.13	0.10	0.10	0.02	0.460
Total	4.62	3.96	3.99	0.50	0.397

*Mean values without common superscript (a and b) differ significantly among CG, AH, and BP groups (p < 0.05)*.

The composition of milk fatty acids was shown in Table 7. Compared with the CG group, the content of saturated fatty acids (SFA) in the BP group goat's milk decreased, and the content of unsaturated fatty acids (UFA) increased. C17:0 in BP group was lower than the CG group (*p* < 0.05). The content of C18:3n−3 in the BP group was higher than that of the CG group and AH group (*p* < 0.05).

**Table 7:** Fatty acids composition in the milk.

**Fatty acids (μg/g)**	**CG**	**AH**	**BP**	**SEM**	***P*-value**
C16:0	57.80	67.90	54.70	4.51	0.060
C17:0	72.40^a^	70.80^ab^	69.80^b^	0.68	0.024
C18:1n−9c	116.60	148.00	163.00	14.76	0.050
C18:2n−6t	51.10	56.20	68.90	5.99	0.059
C18:3n−3	83.00^b^	92.40^b^	104.80^a^	2.60	<0.01
C18:3	198.00	179.80	200.40	7.87	0.076

*Mean values without common superscript (a and b) differ significantly among CG, AH, and BP groups (p < 0.05)*.

### Changes in Related Hormones During Lactation

**Figure 2:**
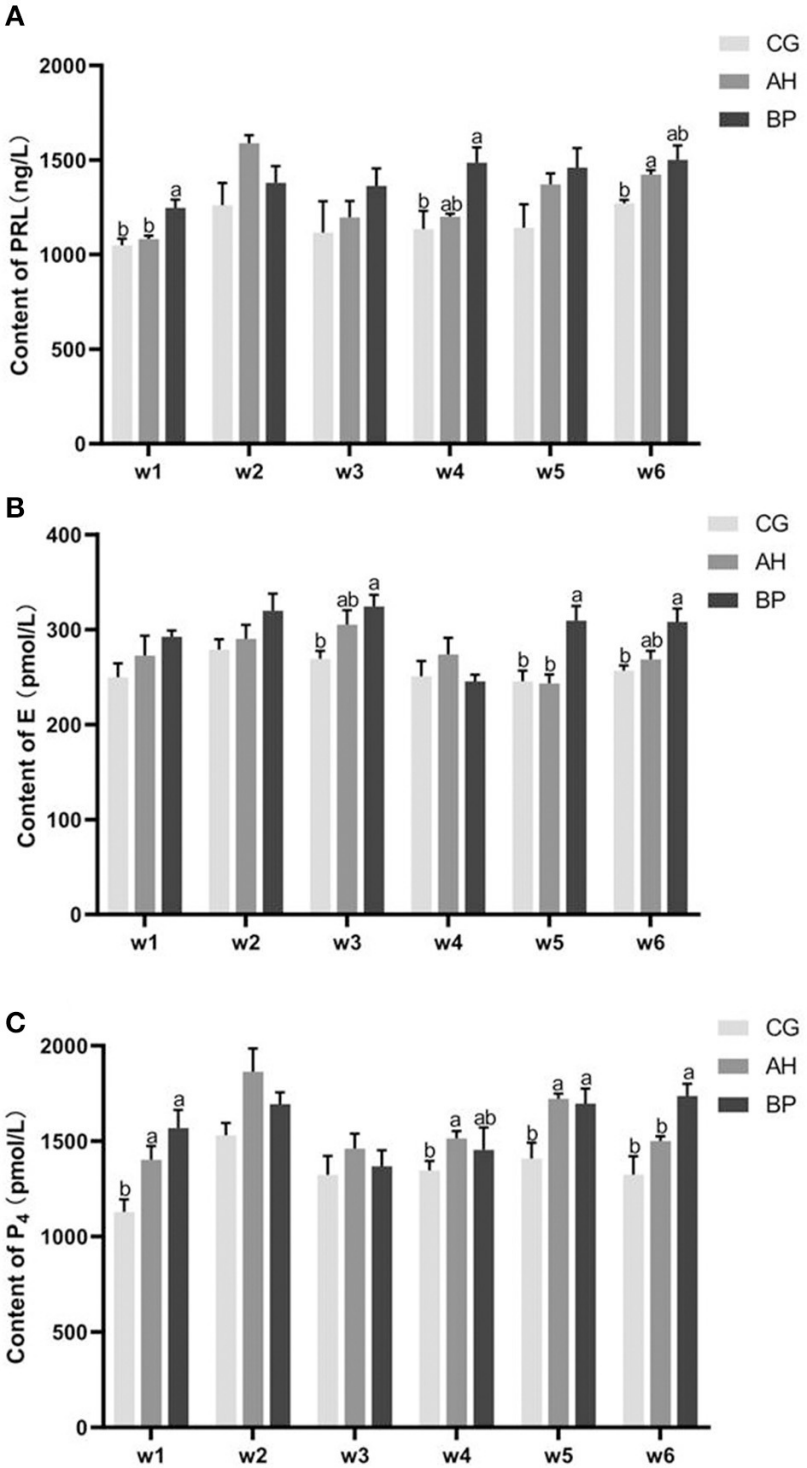
The changes in related hormones during lactation. w1–w6 represents the 1st week to the 6th week. **(A)** PRL (prolactin); **(B)** E (estrogen); **(C)** P_4_ (progesterone). Mean values without common superscript (a and b) differ significantly among CG, AH, and BP groups (*p* < 0.05) and only represent the difference between different groups at the same time point.

As shown in Figure 2, in the 6th week, the PRL content in the serum of the AH group was higher than that in the CG group (*p* < 0.01). In the 1st week (*p* < 0.05) and the 4th week (*p* < 0.05), the PRL content of the BP group was higher than that of the CG group. The level of PRL in the serum of BP group was higher than that of AH group at 1st week (*p* < 0.05; Figure 2A).

In the 3rd week (*p* < 0.05), the 5th week (*p* < 0.05), and the 6th week (*p* < 0.05), the E content of the BP group was higher than that of the CG group. In the 5th week the E content of the BP group was higher than that of the AH group (*p* < 0.05; Figure 2B).

In the 1st week (*p* < 0.05), the 4nd week (*p* < 0.05) and the 5th week (*p* < 0.05), the level of P_4_ in the serum of the AH group was higher than that of the CG group. In the 1st week (*p* < 0.05), the 5th week (*p* < 0.05) and the 6th week (*p* < 0.05), the content of P_4_ of the BP group was higher than that of the CG group. In the 6th week, the P_4_ content in the BP group was higher than that in the AH group (*p* < 0.05; Figure 2C).

### Effect of Alfalfa Flavone and Hybrid *Broussonetia papyrifera* Flavone on the Viability of GMECs

**Figure 3:**
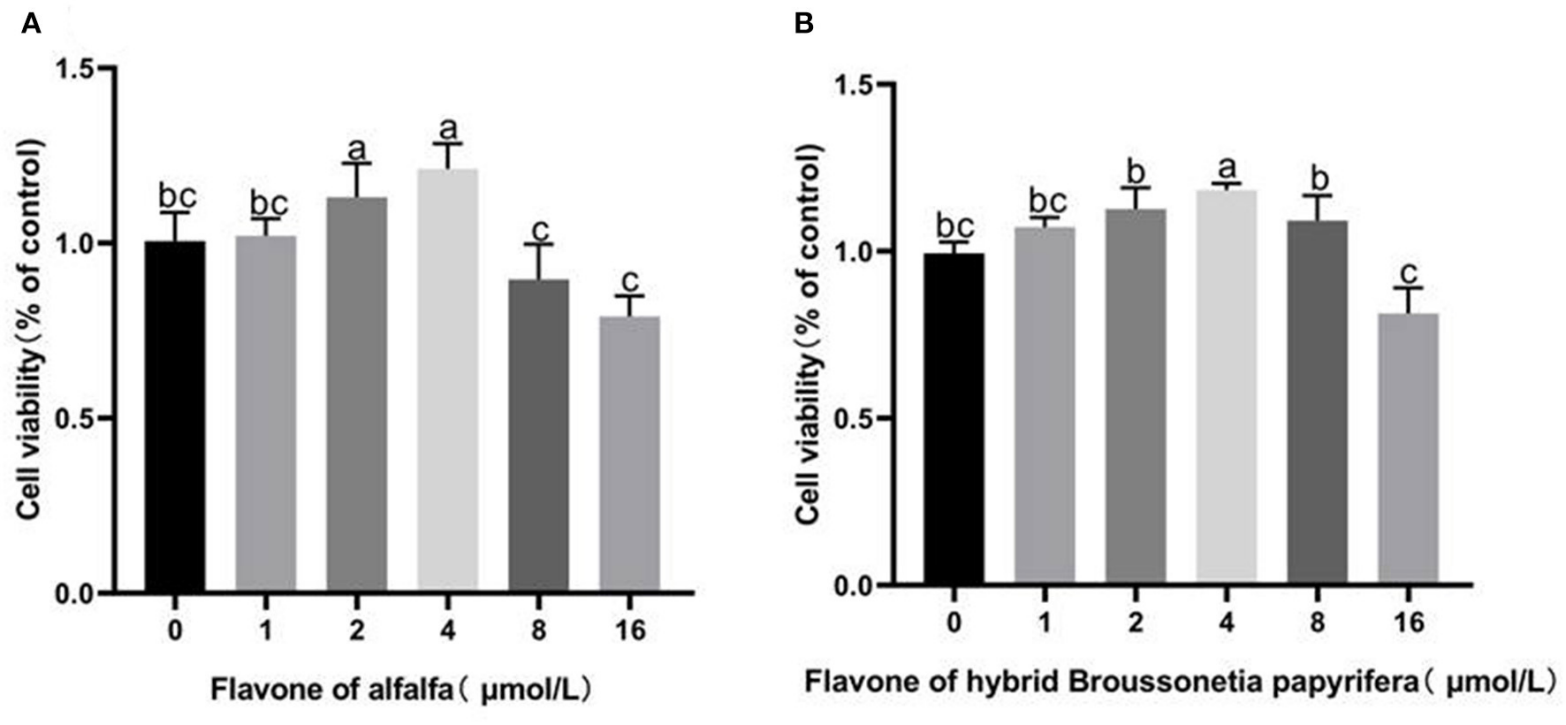
Effect of alfalfa flavone and hybrid *Broussonetia papyrifera* flavone on the viability of gMECs. **(A)** Alfalfa flavone; **(B)** Hybrid *Broussonetia papyrifera* flavone. Mean values without common superscript (a, b, and c) differ significantly among CG, AH, and BP groups (*p* < 0.05).

As shown in Figure 3, adding 1–4 μmol/L alfalfa flavone to the medium and incubating for 48 h increased cell viability. The cell viability reached the peak value at 4 μmol/L treatment, which was higher than that of the CG group (*p* < 0.05). Adding 1–8 μmol/L hybrid *B. papyrifera* flavone to the medium and incubating for 48 h increased cell viability. Similarly, the cell viability at 4 μmol/L treatment reaches the peak value (*p* < 0.01). High concentrations of alfalfa flavone and hybrid *B. papyrifera* flavone (8 and 16 μmol/L) both inhibited cell viability.

### Effect of Alfalfa Flavone and Hybrid *Broussonetia papyrifera* Flavone on TAG Content in GMECs

**Figure 4:**
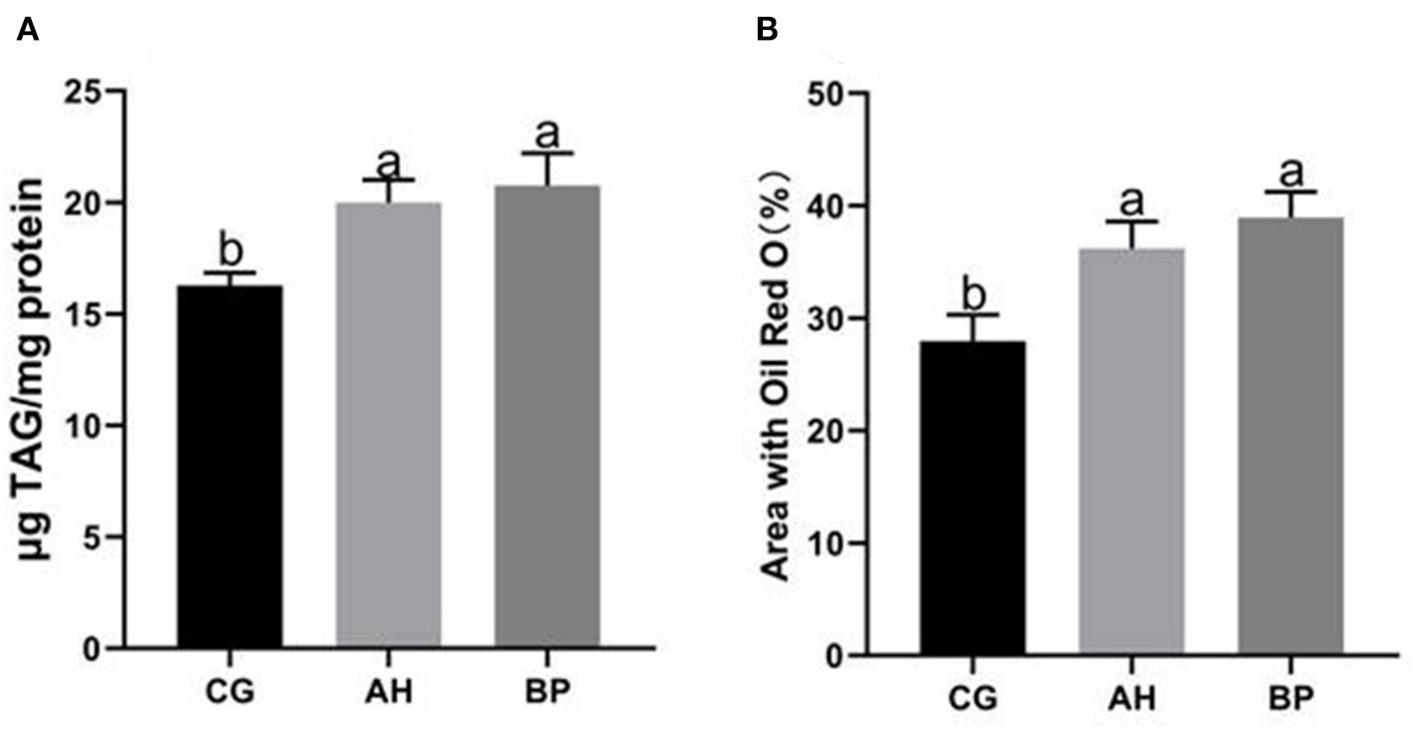
Effect of alfalfa flavone and hybrid *Broussonetia papyrifera* flavone on TAG content in gMECs. **(A)** The TAG content in gMECs; **(B)** Percentage area covered with Oil Red O staining as measured using ImageJ software. CG: control group. AH: the medium contains 4 μmol/L of alfalfa flavone; BP: the medium contains 4 μmol/L of hybrid *Broussonetia papyrifera* flavone. Mean values without common superscript (a and b) differ significantly among CG, AH, and BP groups (*p* < 0.05).

**Figure 5:**
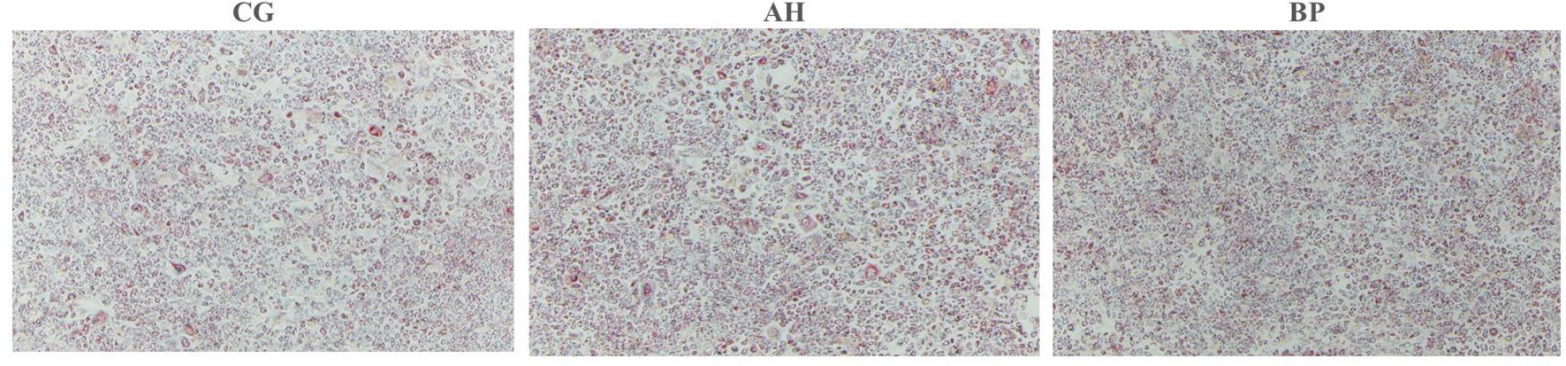
Effect of alfalfa flavone and hybrid *Broussonetia papyrifera* flavone on lipid droplet formation in gMECs. Images were captured at the same spot (40 ×magnification).

Four micromole per liter alfalfa flavone (AH group) and hybrid *B. papyrifera* flavone (BP group) were incubated with gMECs for 48 h, both of which increased the accumulation of intracellular TAG. The TAG content of the AH (*p* < 0.05) group and BP group (*p* < 0.01) was higher than that of the CG group (Figure 4A). Oil red O staining (Figure 5) further confirmed that the number of lipid droplets in gMECs of AH group and BP group was increased (*p* < 0.01), but there was no difference between the AH and BP groups (Figure 4B).

### Effect of Alfalfa Flavone and Hybrid *Broussonetia papyrifera* Flavone on the MRNA of Fatty Acid Synthesis Related Genes in GMECs

**Figure 6:**
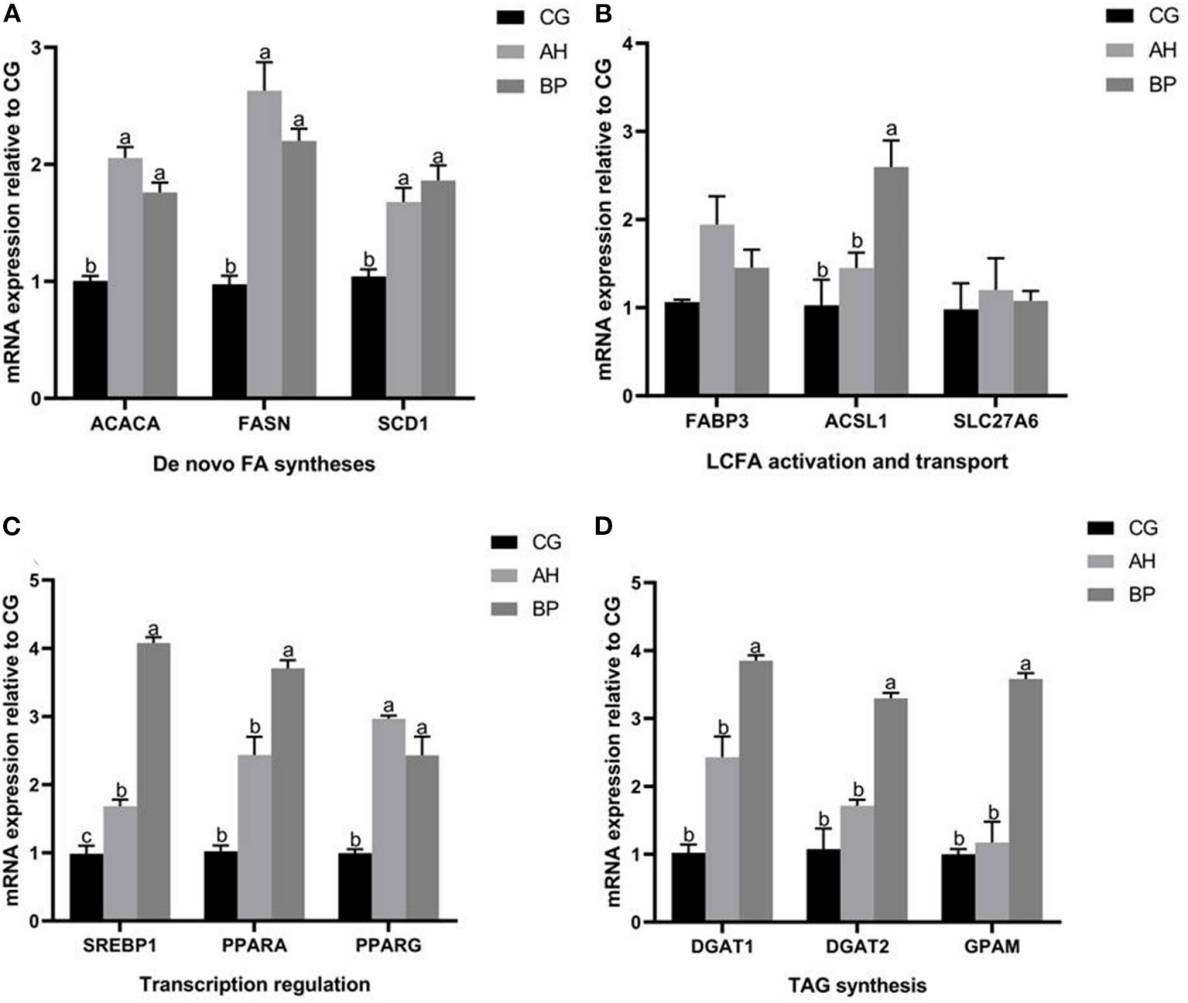
Effect of alfalfa flavone and hybrid *Broussonetia papyrifera* flavone on the mRNA of fatty acid synthesis related genes in gMECs. **(A)** *De novo* FA synthesis; **(B)** LCFA activation and transport; **(C)** Transcription regulation; **(D)** TAG synthesis. Mean values without common superscript (a and b) differ significantly among CG, AH, and BP groups (*p* < 0.05). The c value indicates the mRNA level of SREBP in the BP group was significantly higher than that in the CG and AH groups, and the mRNA level of SREBP in the AH group was significantly higher than that in the CG group.

As shown in Figure 6, the expression of genes related to fatty acid *de novo* synthesis (*ACACA, FASN*, and *SCD1*) in gMECs of AH group and BP group were up-regulated (*p* < 0.05; Figure 6A). Similarly, 4 μmol/L alfalfa flavone and hybrid *B. papyrifera* flavone also promoted the expression of long-chain fatty acid activation and transport related genes, especially the mRNA levels of *ACSL1* was increased of BP group (*p* < 0.01; Figure 6B). The mRNA level of *SREBP1* in the BP group was higher than that in the CG group and AH group (*p* < 0.05), the expression of *SREBP1* in the AH group also increased (*p* < 0.05). The mRNA levels of *PPARA* and *PPARG* of AH group and BP group was increased (Figure 6C). The changes in the expression of the three genes related to TAG synthesis (*DGATI, DGAT2*, and *GPAM*) showed the same trend, and they were all increased by hybrid *B. papyrifera* flavone, which was higher than the CG group (*p* < 0.05) and the AH group (*p* < 0.05; Figure 6D).

### Effect of Alfalfa Flavone and Hybrid *Broussonetia papyrifera* Flavone on the Expression of Hormone Receptor Protein in GMECs

**Figure 7:**
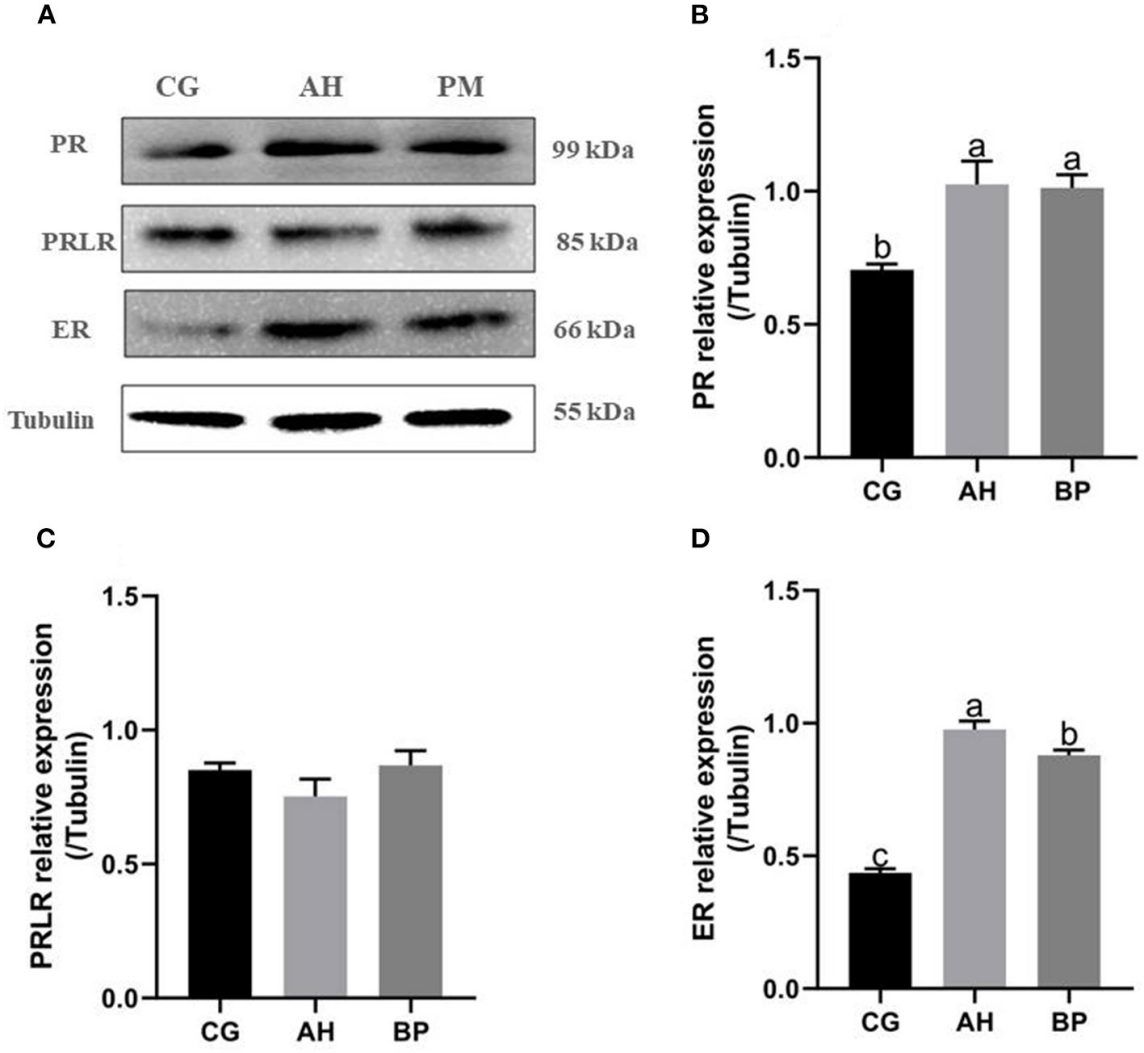
Effect of alfalfa flavone and hybrid *Broussonetia papyrifera* flavone on the expression of hormone receptor protein in gMECs. **(A)** The level of the PR, PRLR, and ER proteins expression in gMECs; **(B)** Protein expression of PR; **(C)** Protein expression of PRLR; **(D)** Protein expression of ER. Mean values without common superscript (a and b) differ significantly among CG, AH, and BP groups (*p* < 0.05). The c value indicates the mRNA level of SREBP in the BP group was significantly higher than that in the CG and AH groups, and the mRNA level of SREBP in the AH group was significantly higher than that in the CG group.

Western Blot results (Figure 7) showed that the expression of progesterone receptor (PR) and estrogen receptor (ER) in gMECs of AH group and BP group was increased. Among them, the expression of PR in the AH and BP groups was higher than that in the CG group (*p* < 0.01). For the expression of ER, the AH group was higher than the CG and BP groups (*p* < 0.01), and the expression of the BP group was higher than CG group (*p* < 0.01). But the expression of prolactin receptor (PRLR) did not change (*p* > 0.05).

## Discussion

Hybrid *B. papyrifera* is a plant rich in biological activities, such as phenolic aldehydes and flavonoids, which are beneficial to animal health after being made into feed (30). Previous studies have shown that the effectiveness and the palatability of feeding mulberry leaves after silage fermentation treatment has been greatly improved (31). In contrast, alfalfa hay has lower moisture content, resulting in lower feed intake. The energy taken by the lactating ewes was mainly used for milk production rather than weight gain (32). In the early lactation of dairy goats, the high energy requirements of milk production and relatively low dry matter intake can lead to negative energy balance (33). Maybe the hybrid *B. papyrifera* fermented feed can increase the DMI of dairy goats, thereby reducing the incidence of diseases caused by negative energy balance.

After the hybrid *B. papyrifera* feed is fermented, it can increase the effective degradation rate of dry matter and the utilization of nutrients, leading to an increase in milk production (34). Hybrid *B. papyrifera* fermented feed increases the peak milk production and slows down the decline in milk production, which can bring more benefits to the farm. Milk production and milk fat percentage generally show a negative correlation (35). The biggest influence on the milk fat rate is the crude fiber content in the feed. If the ratio of concentrate in the diet is too large, the reduction of fiber content will result in a decrease in the ratio of acetic acid/propionic acid, which will eventually lead to a decrease in milk fat rate (36). The level of crude fiber in the BP group's diet especially the content of acid detergent fiber (ADF) is higher, which is helpful to increase the milk fat rate. Crushed feed is too fine to reduce the content of crude fiber, which will eventually lead to a decrease in milk fat rate. Therefore, it is not advisable to add too much concentrate to ruminant feed (17).

UFA in goat milk can reduce the occurrence of chronic diseases such as cardiovascular disease (37). On the contrary, SFA can increase the risk of human disease. Therefore, high-quality dairy products should have high levels of unsaturated fatty acids and low levels of saturated fatty acids. Hybrid *B. papyrifera* fermented feed has a positive effect on the fatty acid composition of goat milk. This is consistent with Si's research (38) whose experimental results show that adding 10–15% of *B. papyrifera* silage in the diet can increase the concentration of polyunsaturated fatty acids in the milk. There are two sources of protein in milk: one is transferred from serum protein; the other is synthesized *de novo* by the absorption of free amino acids from the blood by MECs, and the second way accounts for more than 90% (39). Adding hybrid *B. papyrifera* fermented feed to the diet has no significant difference in the amino acid content of milk, which may be related to the insignificant difference in milk protein and lactose content. Some of the sugar-generating amino acids in the mammary gland, including methionine and glycine, can be used to form lactose, and the rest are all exists in the form of milk protein (40).

The growth and development of mammary glands and the initiation and maintenance of lactation depend on the regulation of various related endocrine hormones (41). P_4_ and E are generally higher during pregnancy, while PRL is higher during lactation (42). PRL plays an important role in regulating breast growth, milk production, and secretion (43). The hybrid *B. papyrifera* fermented feed may regulate milk production through the action of PRL. In most cases, PRL cooperates with other hormones to regulate various functions of the breast, including E, P_4_, and so on (44). Low-dose E can promote lactation in dairy cows, dairy goats and other mammals. During pregnancy, E can cooperate with PRL to promote the development of mammary gland (45). P_4_ and PRL can act on the mammary glands separately, and they can act synergistically to promote the growth and development of the mammary glands during estrus and pregnancy (46). P_4_ may stimulate the pituitary gland to secrete PRL, or may increase the response of mammary epithelial cells to PRL to promote breast development together with it (47). The dairy goats in this experiment are in the transitional stage from full lactation to mid-lactation, and hybrid *B. papyrifera* fermented feed may have a greater impact on the hormone levels in mid-lactation.

We speculated that the regulation of hybrid *B. papyrifera* fermented feed on lactation performance might be the effect of flavone in its leaves, so the mammary epithelial cells were isolated and flavone were added to the culture medium for *in vitro* culture. In this study, high concentration of flavone may exceed the tolerance of the cells. Adding the total flavonoids extracted from geranium to the culture medium of rat intestinal epithelial cells causes the cell viability to increase first and then decrease with the increase of the concentration of flavonoids. The cell viability decreased significantly as the flavone concentration reached 40–50 μg/mL (48). Flavone is a typical antioxidant, which can protect cells, reduce oxidative damage, and enhance cell viability.

Flavone is a powerful antioxidant that exists in nature and contain the structure of 2-phenylchromone. Studies have shown that adding antioxidants such as curcumin to the diets of dairy goats can make the total antioxidant capacity of goat milk higher and lower lipid peroxidation, thereby improving the quality of goat milk (49). Hybrid *B. papyrifera* flavone may have a similar effect, which can promote the mutual conversion of nutrients and increase the synthesis of milk fat. Cytoplasmic lipid droplet's is the direct precursors of milk lipids and are the storage place for newly formed lipids and other neutral lipids (50). The epithelial cells that secrete milk in the mammary gland have the function of synthesizing and secreting large amounts of neutral lipids, which are the main macronutrients in most mammalian milk.

The process of milk fat synthesis includes the *de novo* synthesis of fatty acids, the uptake and activation of fatty acids, the intracellular transport of fatty acids, the extension of fatty acids, the desaturation of fatty acids, the synthesis of TAG, and the formation of lipid droplets (51). Acetyl-CoA is catalyzed by ACACA to synthesize malonyl-CoA. Under the catalysis of FASN, acetyl-CoA (or a small amount of butyryl-CoA) and malonyl-CoA Acyl-CoA condenses, adding two carbon atoms to the carboxyl end of the fatty acid. ACACA is the rate-limiting enzyme. *SCD1* is a key regulatory gene that catalyzes the synthesis of monounsaturated fatty acids (52). Alfalfa flavone and hybrid *B. papyrifera* flavone promote the synthesis of fatty acids through these steps. Melatonin, which is also an antioxidant, can up-regulate the mRNA expression levels of genes related to adipogenesis, lipolysis, β-oxidation, and mitochondrial biosynthesis in pig oocytes (53). Flavone played a similar role on gMECs. Long-chain fatty acids need to pass through the cell plasma membrane to exert their physiological effects. ACSL1 activates LCFA, binds to acyl-CoA (CoA), and then is used to synthesize TAG. FABP3 promotes the cytoplasmic transport of long-chain saturated fatty acids and unsaturated fatty acids. *SLC27A6* is a member of the *SLC27* family, which can promote the absorption of long-chain fatty acids by cells (54). SREBP1 is a key transcription factor for milk fat synthesis. *PPAR* is a ligand-activated receptor superfamily and a nuclear transcription factor, including PPARα and β/δ, γ three subtypes (55). PPARα mainly regulates the β-oxidation of fatty acids (56). It can be speculated that flavone mainly promote the levels of fatty acids by promoting transcriptional regulation. Phosphoglycerol first reacts with a molecule of fatty acyl-CoA to generate 1-fatty acylglycerol-3-phosphate, that is, lysophosphatidic acid (LPA), and the catalyzed enzyme is GPAM. LPA forms phosphatidic acid with another acyl-CoA, and phosphatidic acid phosphatase can remove the phosphate group of phosphatidic acid to obtain 1,2-diacylglycerol (DAG). DAG generates TAG with fatty acyl-CoA. This step is catalyzed by DGAT. This enzyme has two isoenzymes, namely DGAT1 and DGAT2 (57). Flavone played an important role in each step of TAG synthesis, thereby promoting the increase the content of PUFA in goat milk.

Generally speaking, hormones transmit metabolic information by binding to receptors on target cells. Estrogen generally works through its receptor ER, while progesterone signals work through PR (58). It has been reported that flavonoids can directly bind to ERα in animals, and then produce estrogen-like activity (59). Alfalfa flavone and hybrid *B. papyrifera* flavone have the biological activity of estrogen, which led to the increase of estrogen content. PR is regulated by estrogen, and its synthesis in normal MECs requires the combined action of estrogen and ER (60). However, flavone do not work through PRLR to increase the level of prolactin, the specific mechanism is unclear and require further study.

All in all, hybrid *B. papyrifera* fermented feed can increase the milk production of dairy goats while maintaining the stability of the milk composition. It affects milk production by increasing the content of PRL, E, and P_4_ in the serum of goats. Experiments *in vitro* showed that the flavone of the hybrid *B. papyrifera* can promote the synthesis of TAG in gMECs, mainly by increasing the mRNA levels of fatty acid synthesis-related genes. Flavone also significantly increased the protein expression of ER and PR.

## Data Availability Statement

The original contributions presented in the study are included in the article/supplementary material, further inquiries can be directed to the corresponding author.

## Ethics Statement

All animal experiments were approved by the Animal Protection and Utilization Committee of Northwest Agriculture and Forestry University (Yangling, Shaanxi).

## Author Contributions

JiaH, ZW, and MZ conceived and designed experiments. MZ, JinH, YH, XL, and BR conducted experiments. MZ and DL analyzed the data and wrote the manuscript. All authors revised the manuscript and approved the submitted version.

## Funding

This research was supported by Shaanxi Provincial Key Research and Development Program (2019ZDLNY01-03), Shaanxi Province Agricultural Science and Technology Innovation Driven Project (NYKJ-2019-YL19).

## Conflict of Interest

The authors declare that the research was conducted in the absence of any commercial or financial relationships that could be construed as a potential conflict of interest.

## Publisher's Note

All claims expressed in this article are solely those of the authors and do not necessarily represent those of their affiliated organizations, or those of the publisher, the editors and the reviewers. Any product that may be evaluated in this article, or claim that may be made by its manufacturer, is not guaranteed or endorsed by the publisher.
